# Antiviral Activity of *Bacillus* sp. Isolated from the Marine Sponge *Petromica citrina* against Bovine Viral Diarrhea Virus, a Surrogate Model of the Hepatitis C Virus

**DOI:** 10.3390/v5051219

**Published:** 2013-04-29

**Authors:** Juliana Cristina Santiago Bastos, Luciana Konecny Kohn, Fabiana Fantinatti-Garboggini, Marina Aiello Padilla, Eduardo Furtado Flores, Bárbara Pereira da Silva, Cláudia Beatriz Afonso de Menezes, Clarice Weis Arns

**Affiliations:** 1 Laboratório de Virologia, Departamento de Genética Evolução e Bioagentes, Instituto de Biologia/Universidade Estadual de Campinas-UNICAMP, Cx. Postal 6109, CEP 13083-970, Campinas, SP, Brazil; E-Mails: lucianakohn@gmail.com (L.K.K.); fabianaf@cpqba.unicamp.br (F.F.-G.); ma_padilla2001@yahoo.com.br (M.A.P.); barbarapds84@gmail.com (B.P.d.S); clbeatriz2003@gmail.com (C.B.A.d.M.); clarns@gmail.com (C.W.A.); 2 Centro de Ciências Rurais, Departamento de Medicina Veterinária Preventiva. Universidade Federal de Santa Maria (UFSM) CEP 97105-900 - Santa Maria, RS, Brazil; E-mail: eduardofurtadoflores@gmail.com

**Keywords:** hepatitis C virus, antiviral agents, marine microorganisms

## Abstract

The Hepatitis C virus causes chronic infections in humans, which can develop to liver cirrhosis and hepatocellular carcinoma. The Bovine viral diarrhea virus is used as a surrogate model for antiviral assays for the HCV. From marine invertebrates and microorganisms isolated from them, extracts were prepared for assessment of their possible antiviral activity. Of the 128 tested, 2 were considered active and 1 was considered promising. The best result was obtained from the extracts produced from the *Bacillus* sp. isolated from the sponge *Petromica citrina.* The extracts 555 (500 µg/mL, SI>18) and 584 (150 µg/mL, SI 27) showed a percentage of protection of 98% against BVDV, and the extract 616, 90% of protection. All of them showed activity during the viral adsorption. Thus, various substances are active on these studied organisms and may lead to the development of drugs which ensure an alternative therapy for the treatment of hepatitis C.

## 1. Introduction

Viruses cause many important diseases, and viral-induced emerging and re-emerging infectious diseases representing a major health threat to the public. Effective control of viral infection has remained an unachieved goal, due to virus intracellular replicative nature and readily mutating genomes, as well as the limited availability of anti-viral drugs and measures [1].

Hepatitis C virus (HCV) infection is a serious global health problem, and the patients with chronic infection have risk of developing liver cirrhosis and hepatocellular carcinoma [2]. HCV infects as many as 170 million people worldwide, representing 3% of the world’s population [3]. The big problem is that, in general, people with chronic hepatitis C are relatively asymptomatic and have few if any clinical manifestations prior to the development of cirrhosis [4].

Currently, no vaccine exists for hepatitis C vírus [5]. The treatment for HCV infection consists of pegylated interferon (IFN)-α in combination with the nucleoside analog ribavirin (1-β-D-ribofuranosyl-1,2,4-triaxole-3-carboxamide) [6]. This therapy is expensive, effective in only a subset of patients and associated with many side effects [7] such as depression, flu-like symptoms, fatigue, and hemolytic anemia, and, due to this, many patients are forced to discontinue therapy [8].

Since the discovery of the HCV, its propagation in cell culture has been a major goal for virologists worldwide. All human hepatitis viruses are very difficult to grow in cell culture [9]. Because of this, another virus is used as a surrogate model for studies regarding HCV.

The bovine viral diarrhea virus (BVDV), a member of the *Flaviviridae* family (genus *Pestivirus*), shares similarities with HCV (genus *Hepacivirus*, *Flaviviridae* family) in terms of their replication cycles, biology and genetic organization, and shows the functionally homologous nature of many of their gene products, which are considered to be major targets for the development of anti-HCV agents [10]. 

BVDV is easy to culture *in vitro*, molecular clones are available for genetic studies and the virus undergoes a complete replication cycle. Both BVDV and HCV utilize the LDL receptor to enter cells, use a functionally similar internal ribosome entry site for translation, have a mechanistically similar NS5B RNA-dependent RNA polymerase, and a seemingly equivalent mechanism of virion maturation, assembly and egress [11].

For these reasons, BVDV is considered to be a valuable surrogate virus model for identifying and characterizing antiviral agents for use against HCV [11,12,13,14,15]. 

Because of the lack of available vaccines and treatments not being tolerated by some patients, new antiviral agents to treat HCV infection are desperately needed [11].

Natural products derived from terrestrial and marine kingdoms represent an inexhaustible source of compounds with promising antiviral action, mainly for the variety of synthesized metabolites. In relation to infectious diseases, the exploration of the marine environment represents a promising strategy in the search for active compounds. It is necessary due to the appearance of resistance to available treatments in many microorganisms [1].

In this study, extracts were tested against BVDV to evaluate their potential antiviral activity.

## 2. Materials and Methods

### 2.1. Crude Extracts

Extracts derived from the metabolism of the bacteria strain were obtained by the method of liquid-liquid separation. A pre-inoculum of the isolates were cultured in 7 mL of isolation medium and incubated at 30 °C for 24 h at 100 rpm. After growth of the culture, the total volume was transferred to an Erlenmeyer flask containing 50 mL of the same medium and kept at the same temperature and rotation for 24 h. Again, 50 mL of bacterial growth were transferred to a glass jar containing 500 mL of the same medium and kept in the same conditions mentioned for seven days. After this period, 500 mL of ethyl acetate were added and the mixture was triturated in a blender with high rotation. Then, the mixture was stirred at 100 rpm for 24 h.

Initially, the mixture was filtered on Buchner funnel containing a pad of celite and then the organic phase was recovered in an Erlenmeyer flask using a separatory funnel. In the aqueous phase, the cell debris and the culture medium were retained, and in the organic phase, the possible biologically active metabolites were recovered.

The organic phase was then filtered through cotton and transferred to a round bottom flask. Extracts contained in the organic phase were concentrated in rotaevaporador (BuchiRotavapor R-215) in vacuum at 37 °C until complete drying of the solvent. Then the extracts were suspended in methyl alcohol, filtered with cotton, and the volume transferred to new tubes glass using Pasteur pipette. These tubes were led to Savant vacuum centrifuge, model 210A Speedvac ® Plus SC for evaporation. After complete drying of the solvent, the extracts were dissolved in 10% DMSO in medium AMH and kept at 4 °C until use in the antiviral activity assays.

### 2.2. Antiviral Activity Assay

#### 2.2.1. Virus and cell lines.

The Madin–Darby bovine kidney (MDBK) cell were propagated in monolayer cultures using minimal essential medium (MEM) with Earles’s salts, supplemented with 10% equine serum. Cells were passaged at 1:2 to 1:10 dilutions according to conventional procedures using 0.05% trypsin with 0.02% EDTA.

#### 2.2.2. Cell cytotoxic effect

100 μL of suspension per well were seeded in 96-well culture plates at a density of 1 x 10^5^ cells/mL. The microtiter plates containing cells were pre-incubated for 24 h at 37 °C to allow stabilizations prior to addition (100 μL) of samples at 11 concentrations (500 to 0,48 μg/mL). The maximum nontoxic concentration (MNTC) was determined microscopically by the observation of morphological changes of cells at 24, 48 and 72 h of incubation, and after 72 h the results were obtained with MTT assay.

#### 2.2.3. MTT assay

The MTT assay is a sensitive *in vitro* assay for the measurement of cell proliferation or a reduction in cell viability. Cells were cultured in 96-well tissue culture plates. The tetrazolium compound MTT (3-[4, 5-dimethylthiazol-2-yl]-2, 5-diphenyltetrazolium bromide) was added to the wells and the cells were incubated. MTT is reduced by metabolically active cells to insoluble purple formazan dye crystals. Dimethylsulfoxide was then added to the wells, solubilizing the crystals so the absorbance could be read using a spectrophotometer on absorbance of 540 nm [16,17]. The data was analyzed by plotting cell number versus absorbance, allowing quantitation of changes in cell proliferation. The rate of tetrazolium reduction is proportional to the rate of cell proliferation and indirectly indicates the reduction in cell viability caused by the action of virus.

#### 2.2.4. Titration of viruses

The cells were seeded in 96-well culture plates at a density of 1 x 10^5^ cells/mL and then incubated at 37 º C in a humidified atmosphere containing CO_2_ for 24 h. Serial dilutions of virus stocks were prepared and cells were infected with the dilution of virus. After an additional incubation (1–2 days), the cytopathic effect was recorded. The 50% tissue-culture infective dose (TCID_50_) per mL was calculated as described previously by Reed and Münch [18].

#### 2.2.5. Antiviral activity

Determination of antiviral activity was based on cytopathic effect inhibition. All experiments were performed in quadruplicate. Briefly, for evaluation of inhibition, the cells were seeded in 96-well culture plates. After 24 h of incubation, the medium was replaced with 100 µL of MEM(E) containing the extracts at 50 µg/mL, and 50 µL of 100 TCID_50_/50µL of viruses were added in quadruplicate and incubated for 3 days. Controls consisted of untreated infected (virus at 100 TCID_50_/50µL), treated noninfected (extract control), and untreated noninfected. The cytophatic effect was observed after 72 h and the extracts with antiviral activity were determinate. For quantification of this activity, the MTT assay was performed. This assay allows the quantification of cell viability and indirectly allows quantification of cell extract protection against the virus.

The protection percentage was calculated using the following formula [19]: (Absorbance of treated – absorbance of the control virus) / (Absorbance of the cellular control - absorbance of the control virus) X 100.

The test of antiviral activity was evaluated initially with a single dose at MNTC against 100 TCID_50_/mL of virus. The extracts with activity greater than 90% were considered promising. 

To confirm activity, a concentration response curve with different concentrations of extract in the presence of 100 TCID_50_/mL of virus was determined by MTT assay, to determine antiviral concentration 50% (EC_50_). 

The EC_50_ were calculated from concentration-effect curves after linear regression analysis. The results were obtained from triplicate assays with at least five extract concentrations. The percentage of cytotoxicity was calculated as [(A – B)/A] x 100, where A and B are the OD540 of untreated and of treated cells, respectively. The percentages of protection were calculated as [(A − B) × 100/(C − B)], where A, B and C indicate the absorbance of the extracts, virus and cell controls, respectively. Each obtained EC_50_ value was defined as the effective concentration that reduced the absorbance of infected cells to 50% when compared with cell and virus controls. The 50% inhibition (IC_50_) for each compound were obtained from dose-effect-curves (not shown) generated by plaque assay after linear regression analysis. The EC_50_ and IC_50_ are the average of three assays with five concentrations within the inhibitory range of the compounds. The therapeutic index (*i.e.,* selective index) was defined as EC_50_/IC_50_.

#### 2.2.6. The potential stage in the viral infection cycle

Cells and viruses were incubated with active extracts at different stages during the viral infection cycle in order to determine the mode of antiviral action. Cells were pretreated with samples before viral infection; viruses were incubated with samples before infecting the cells; and the cells were infected with the virus and incubated together before the addition of samples. Samples were used at the maximum noncytotoxic concentration. Subsequent assays were performed to differentiate the active extracts in the pretreatment between adsorption and penetration.

Values were expressed as titer (TCID_50_/µL) and inhibition percentage (IP) as described in [20]. The inhibition percentage was calculated by the formula: (IP) = (1 – T/C) x 100, where T is the antilog of the extract-treated viral titers and C is the antilog of the control (without extract) viral titers. IP was considered positive at greater than or equal to 98%.

#### 2.2.7. Virucidal action

In order to evaluate possible extracellular viral inactivation by extracts, equal volumes (100 µL) of the 10-fold serially diluted virus suspension and MNTC of the extracts were mixed and incubated for 1h at 37 °C. Each mixture was then added to the cell monolayer, and infectious titers were compared with controls.

### 2.3. Statistical analysis

The results were expressed as mean ± s.e.m. The selectivity index (SI) was determined by the ratio of CC_50_ to EC_50_. The statistically different effects of tested extracts on the inhibition of virus replication were compared with the control group using the Student’s t-test with p≤0.05 for significant result. 

### 2.4. Identification of bacteria by 16S rRNA gene sequencing analysis

One sample of each of the sponges *Petromica citrina* (PC), and *Chelonaplysilla erecta* (CE) were collected at Saco do Poço (23453S; 45158W), and Ilha de Serraria (23484S; 45134W) at Ilha Bela region, São Paulo State, Brazil, at depths between 5 and 15 m. The sponges were placed in sterilized polyethylene bags containing seawater and immediately transported to the Centro de Biologia Marinha (CEBIMar) of the Universidade de São Paulo. Sponge samples were washed twice with sterilized seawater, cut into pieces and then thoroughly homogenized in a sterile mortar with sterile seawater. Triturated samples were diluted in hundred-fold series (10^-2^, 10^-4^) and aliquots of 100 µL were inoculated into Petri dishes containing the media: TSA Agar (Difco, USA), Marine Agar (DifcoTM, USA) and M1 (soluble starch 10 g/L, yeast extract 4 g/L, peptone 2 g/L, agar 15 g/L). All media were prepared with artiﬁcial seawater (ASW): KBr 0.1 g/L, NaCl 23.48 g/L, MgCl_2_.6H_2_O 10.61 g/L ,CaCl_2_ .2H_2_O 1.47 g/L, KCl 0.66 g/L 1, SrCl_2_ .6H_2_O 0.04 g/L, Na_2_SO_4_ 3.92 g/L, NaHCO_3_ 0.19 g/L, H_3_BO_3_ 0.03 g/L. Cycloheximide (50 µg.mL^-1^) and nalidixic acid (15 µg.mL^-1^) were added to inhibit fungal and many fast-growing Gram-negative bacteria, respectively. Agar plates were incubated at 25 ºC and different colonies were isolated from the 2nd to the 30th day of plating. Pure cultures were obtained after serial transfers to the same culture medium used to plate the sponge samples. The maintenance of the isolates was performed by cryopreservation at -80 ºC (10% glycerol).

The strains were cultured in Nutrient Broth (NB) (Difco) for 24-48 h at 28 °C and genomic DNA was extracted from each strain [21]. From the genomic DNA, 16S rRNA gene sequences were ampliﬁed by polymerase chain reaction (PCR) using bacterial universal primers p27f e p1401r [22] and sequencing [23].

## 3. Results

The antiviral activity of each extract was evaluated initially in a standard titer assay where the extracts at nontoxic concentrations were added at the same time as the virus. This initial analysis identified which among the many extracts tested have antiviral activity against the virus. The results were considered promising if they showed a protection percentage of at least 90% and active if the result was higher than 97%.

One hundred twenty-eight extracts were tested, of wich two were active against the virus. This represents 1,5% of the total. One of them was considered promising. The Table 1 summarizes the information about the best results. 

**Table 1:** General characteristics of the extracts evaluated for antiviral activity.

Extract	Sponge	Genbank Access number	Identification	Inhibition percentage (%)
**B511**	*Chelonaplysilla erecta*	HQ433229	*Exiguobacterium* sp.	33
**B515**	*Chelonaplysilla erecta*	HQ433231	*Vibrio* sp.	25
**B555**	*Petromica citrina*	Deposit number in process	*Bacillus* sp.	98
**B565**	*Petromica citrina*	Deposit number in process	*Bacillus* sp.	37
**B584**	*Petromica citrina*	Deposit number in process	*Bacillus* sp.	98
**B616**	*Petromica citrina*	Deposit number in process	*Bacillus* sp.	90

Characteristics of the extracts evaluated for antiviral activity. Name of the each extract evaluated, sponge from which the microorganism was obtained, Genbank access number, identification of the microorganism from which the extract was obtained, and inhibition percentage obtained on antiviral assay

### 3.1. The potential stage in the viral infection cycle

To identify the stage of viral replication cycle on which the active extracts could be operating, three different treatments were carried out. The cells were infected with virus followed by addition of the extract after 1 h, to evaluate the viral replication phase; viruses were pretreated with the extract before infecting the cells (virus inactivation); while addition of the extract to the cells before viral infection was performed to evaluate any effect in terms of viral adsorption. In all experiments the extract was used at its MNTC. The potential stage in the viral infection cycle for each promising and active extract is shown in the table 2.The MNTC, CC_50_, IC_50_ and SI of the active and promising extracts against BVDV were calculated and the results are listed in Table 2.

**Table 2:** Promising and active extracts.

Extract	MNTC (µg/mL)	EC_50_ (µg/mL)	IC_50_ (µg/mL)	SI	Activity
B555	>500	>500	27,35	>18	Pretreatment: Adsorption
B584	150	277	10,24	27	Pretreatment: Adsorption
B616	1000	1500	47	30,6	Pretreatment: Adsorption

Characteristics of the promising and active extracts. The active extracts are 555 and 584, and the promising extract is 584. The maximum nontoxic concentration (MNTC), antiviral concentration 50% (EC_50_), the 50% inhibition (IC_50_), the selective index (SI) and the potential stage in the viral infection cycle of antiviral action.

According to these results, the three extracts showed SI greater than 4, which ensures its viability in *in vitro* assays and all of them act by inhibiting virus adsorption to cell.

## 4. Discussion

The exploration of the marine environment represents a promising strategy in the search for active compounds against infectious diseases [1]. This environment is still underexplored in Brazil, despite its large size and enormous diversity of species. 

Besides its peculiar structures, marine natural products have an extraordinary diversity of molecular targets with expressive selectivity. This greatly increases the pharmacological and therapeutic potential of these molecules [24].

Marine sponges are among the richest sources of pharmacologically active products derived from marine organisms by their secondary metabolites and also by substances produced by their associated microorganisms. There are already known more than 5300 products that represent possible alternatives against diseases of bacterial, viral, fungal and parasitic origin [25].

Bacteria that gave rise to extracts evaluated in this study were isolated from marine sponges *Petromica citrina* and *Chelonaplysilla erecta*. The marine sponge *Petromica citrina* is endemic in the country. Previously, antimicrobial activity against some resistant bacteria from aqueous extracts of this sponge has been demonstrated [26]. However, here, we only isolated active extracts of microorganisms from this sponge. 

From the sponges, bacteria were isolated, *Vibrio* sp. and *Bacillus* sp., and the extracts obtained from them were evaluated against BVDV. Three of these *Bacillus* generated two active extracts (B555 and B584, IP=98%) and one promising (B616, IP=90%) against BVDV. These microorganisms were identified as *Bacillus* sp. These extracts showed SI greater than 4, which ensures its viability on *in vitro* assays.

Several activities have been described in relation to *Bacillus* sp. Studies showed that bacteria of this genus can devote more than 3% of its genome to genes related to the biosynthesis of secondary metabolites [27]. 

The experimental study of antiviral activity of spore-forming bacterium *Bacillus pumilus* "Pashkov", carried out in 2010, showed for the first time effective antiviral activity of cultural fluid that opens perspectives for development of medications against enterovirus infections [28].

The *Bacillus intermedius* RNAse showed antiviral activity in guinea-pigs and rabbits infected with outdoor rabies virus. The intramuscular injection of RNAase failed to protect the infected animals [29]. This *Bacillus intermedius* RNAse previously showed antiviral activity in experiments with mice preinfected with street rabies virus [30].

A ribonuclease (RNase) with tobacco mosaic virus inhibition was isolated and purified from *Bacillus cereus*. The inhibitory activity of the RNase in the purification process against tobacco mosaic virus was tested, and the percentage inhibition of the purified RNase reached 90% [31].

Besides antiviral activity, *Bacillus* species have had several other activities described.

One study shows the Antiplasmodial activity of bacilosarcin A isolated from the octocoral-associated bacterium *Bacillus* sp. collected in Panama. The antiplasmodial activity of the isolated compounds was evaluated *in vitro* against the chloroquine-resistant *Plasmodium falciparum* strain W2 and this study constitutes a new addition to the few existing antiplasmodial metabolites isolated from heterotrophic bacteria associated with corals, showing that bacteria associated with marine invertebrates represents a promising resource for antimalarial research [32].

Another study showed the antibacterial and anticancer activity of ε-poly-L-lysine (ε-PL) produced by a marine *Bacillus subtilis*. The bacterium produced an active compound against a number of gram negative bacteria [33].

Already been demonstrated, yet, antifungal activity of *Bacillus coagulans* against *Fusarium* sp. [34], the activity of Macrolactin S isolated from *Bacillus* sp. AT 28 that inhibited the growth of *S. aureus*, *Bacillus subtilis*, and *Escherichia coli* [35], and the antimicrobial activity of *Bacillus* sp. strain FAS1 isolated from soil was demonstrated on the standard indicator species [36].

Therefore, this genus represents a rich source of bioactive substances and, according to the obtained results, can lead to the development of a new treatment option against HCV infection.

The preliminary tests of mechanism of action showed that the active extracts (B555 and B584) and the promising extract (B616) acts by inhibiting virus adsorption to cell, thus interfering with the progression of infection. This may result from changes in proteins involved in the interaction between the viral envelope and endosomal membrane.

## 5. Conclusion

The results of the present study provide further evidence for the potential of microorganisms isolated from marine invertebrates, that represents a reservoir of pharmacologically active substances. From microorganisms isolated from marine sponges, compounds with potential to lead to the development of an alternative therapy against HCV infection were identified. Two of them provided 98% of protection to the cell and their selectivity index was satisfactory. This activity occurred during the adsorption of the virus to the cell. Thus, the extract was able to interfere with the progression of infection. Further studies are necessary to discover the substance responsible for this activity and this substance may lead to the development of an alternative therapy as needed for the population.

## References

[1] Yasuhara-Bell J., Yang Y., Barlow R., Trapido-Rosental H., Lu Y. (2010). In vitro evaluation of marine microorganism extracts for antiviral activity. Virol. J..

[2] Ravikumar Y.S., Upasana R., Nandhitha M., Perween A., Naika H.R. (2011). Inhibition of hepatitis C virus replication by herbal extract: *Phyllanthus amarus* as a potent natural source. Virus Res..

[3] Li H., Stoddard M.B., Wang S., Blair L.M., Giorgi E.E. (2012). Elucidation of Hepatitis C Virus Transmission and Early Diversification by Single Genome sequencing. PLoS Pathog..

[4] Suzuki T., Ishii K., Aizaki H., Wakita T. (2007). Hepatitis C viral life cycle. Adv. Drug Del. Rev..

[5] Barnes E., Folgori A., Capone S., Swadling L., Aston S., Kurioka A., Meyer J., Huddart R., Smith K., Townsend R., Brown A., Antrobus R., Ammendola V., Naddeo M., O'Hara G., Willberg C., Harrison A., Grazioli F., Esposito M.L., Siani L., Traboni C., Oo Y., Adams D., Hill A., Colloca S., Nicosia A., Cortese R., Klenerman P. (2012). Novel adenovirus-based vaccines induce broad and sustained T cell responses to HCV in man. SciTransl Med..

[6] Sako K., Aoyama H., Sato S., Hashimoto Y., Baba M. (2008). γ-Carboline derivatives with anti-bovine viral diarrhea virus (BVDV) activity. Bioorg. Med. Chem..

[7] Martinot-Peignoux M., Boyer N., Pouteau M., Castelnau C., Giuily N., Duchatelle V., Aupérin A., Degott C., Benhamou J.P., Erlinger S. (1998). Predictors of sustained response to alpha interferon therapy in chronic hepatitis C. J. Hepatol..

[8] Lemon S.M., McKeating J.A., Pietschmann T., Frick D.N., Glenn J.S., Tellinghuisen T.L., Symons J., Furman P.A. (2010). Development of novel therapies for hepatitis C. Antiviral Res..

[9] Duverlie G., Wychowski C. (2007). Cell culture systems for the hepatitis C virus. J. Gastroenterol..

[10] Finkielsztein L.M., Moltrasio G.Y., Caputto M.E., Castro E.F., Cavallaro L.V., Moglioni A.G. (2010). What is known about the antiviral agents active against bovine viral diarrhea virus (BVDV)?. Curr. Med. Chem..

[11] Buckwold V.E., Beer B.E., Donis R.O. (2003). Bovine viral diarrhea virus as a surrogate model of hepatitis C virus for the evaluation of antiviral agents. Antiviral Res..

[12] Zitzmann N., Mehta A.S., Carrouee S., Butters T.D., Platt F.M., McCauley J., Blumberg B.S., Dwek R.A., Block T.M. (1999). Imino sugars inhibit the formation and secretion of bovine viral diarrhea virus, a Pestivirus model of hepatitis C virus: implications for the development of broad spectrum anti-hepatitis virus agents. Proc. Natl. Acad. Sci..

[13] Baginski S.G., Pevear D.C., Seipel M., Sun S.C., Benetatos C.A., Chunduru S.K., Rice C.M., Collett M.S. (2000). Mechanism of action of a pestivirus antiviral compound. Proc. Natl. Acad. Sci..

[14] Buckwold V.E., Wei J., Wenzel-Mathers M., Russell J. (2003). Synergistic in vitro interactions between alpha interferon and ribavirin against bovine viral diarrhea virus and yellow fever virus as surrogate models of hepatitis C virus replication. Antimicrobial. Agents Chemother..

[15] Yanagida K., Baba C., Baba M. (2004). Inhibition of bovine viral diarrhea virus (BVDV) by mizoribine: synergistic effect of combination with interferon-α. Antiviral Res..

[16] Mosmann T. (1983). Rapid colorimetric assay for cellular growth and survival: application to proliferation and cytotoxicity assays. J. Immun. Meth..

[17] Scudiero D.A., Shoemaker R.H., Paull K.D. (1988). Evaluation of a soluble tetrazolium/formazan assay for cell growth and drug sensitivity in culture using human and other tumor cell lines. Cancer Res..

[18] Reed L.J., Münch H.A. (1938). A simple method of estimating fifty percent endpoints. Am. J. Hyg..

[19] Takeuchi H., Baba M., Shigeta S. (1991). An application of tetrazolium (MTT) colorimetric assay for the screening of anti-herpes simplex virus compounds. J. Virol. Methods..

[20] Koseki I., Simoni I.C., Nakamura I.T., Noronha A.B., Costa S.S. (1990). Antiviral activity of plant extracts against aphtovirus, pseudorabiesvírus and pestivirus in cell cultures. Microbios. Letters..

[21] Pospiech A., Neumann B. (1995). A versatile quick-prep of genomic DNA from Gram-positive bacteria. Trends Genetics.

[22] Lane D.J., Pace B., Olsen G.J., Stahl D.A., Sogin M.L., Pace N.R. (1985). Rapid determination of 16S ribosomal RNA sequences for phylogenetic analysis. Proc. Nat. Acad. Sci..

[23] Menezes C.B.A., Bonugli-Santos R.C., Miqueletto P.B., Passarini M.R.Z., Silva C.H.D., Justo M.R., Rebeca R., Fantinatti-Garboggini F., Oliveira V.M., Berlinck R.G.S., Sette L.D. (2010). Microbial diversity associated with algae, ascidians and sponges from the north coast of São Paulo state, Brazil. Microbiol. Res..

[24] Costa-Lotufo L.V., Wilke D.V., Jimenez P.C., Epifanio R.A. (2009). Organismos marinhos como fonte de novos fármacos: Histórico & perspectivas. Quim. Nova..

[25] Laport M.S., Santos O.C., Muricy G. (2009). Marine sponges: potential sources of new antimicrobial drugs. Curr. Pharm. Biotechnol..

[26] Marinho P.R., Muricy G.R.S., Silva M.F.L., de Marval M.G., Laport M.S. (2010). Antibiotic-resistant bacteria inhibited by extracts and fractions from Brazilian marine sponges. Brazilian J. Pharmac..

[27] Donadio S., Monciardini P., Sosio M. (2007). Polyketide synthases and nonribosomal peptide synthetases: the emerging view from bacterial genomics. Nat. Prod. Rep..

[28] Mikhaĭlova N.A., Nagieva F.G., Grin'ko O.M., Zverev V.V. (2010). Experimental study of antiviral activity of spore-forming bacterium *Bacillus pumilus* "Pashkov". Mikrobiol. Epidemiol. Immunobiol..

[29] Gribencha S.V., Potselueva L.A., Barinskiĭ I.F., Deev S.M., Balandin T.G., Leshchinskaia I.B. (2006). Antiviral activity of *Bacillus intermedius* RNAase in guinea-pigs and rabbits infected with outdoor rabies virus. Vopr. Virusol..

[30] Gribencha S.V., Potselueva L.A., Barinskiĭ I.F., Balandin T.G., Deev S.M., Leshchinskaia I.B. (2004). The antiviral activity of RNAse *Bacillus intermedius* in experiments with mice preinfected with street rabies virus. Vopr. Virusol.

[31] Zhou W.W., Niu T.G. (2009). Purification and some properties of an extracellular ribonuclease with antiviral activity against tobacco mosaic virus from *Bacillus cereus*. Biotechnol. Lett..

[32] Boya C.A., Herrera L., Guzman H.M., Gutierrez M. (2012). Antiplasmodial activity of bacilosarcinA isolated from the octocoral-associated bacterium *Bacillus sp*. collected in Panama. J. Pharm. Bioallied. Sci..

[33] El-Sersy N.A., Abdelwahab A.E., Abouelkhiir S.S. (2012). Antibacterial and anticancer activity of ε-poly-L-lysine (ε-PL) produced by a marine *Bacillus subtilis* sp. J. Basic Microbiol..

[34] Craczyc K., Trojanowska K., Mueller A. (2002). Antifungal activity of *Bacillus coagulans* against *Fusarium* sp. Acta Microbiol. Pol..

[35] Sohn M.J., Zheng C.J., Kim W.G. (2008). Macrolactin S, a new antibacterial agent with FabG-inhibitory activity from *Bacillus* sp. AT28. J. Antibiot..

[36] Moshafi M.H., Forootanfar H., Ameri A. (2011). Antimicrobial activity of *Bacillus* sp. strain FAS1 isolated from soil. Pak. J. Pharm. Sci..

